# Small auxin-up RNA promotes *GmMYB176* gene transcription to modulate seed isoflavone accumulation in soybean

**DOI:** 10.3389/fpls.2026.1841460

**Published:** 2026-05-07

**Authors:** Yujia Ji, Xiaofei Ma, Yuecheng Tang, Yuhong Zheng, Yongguo Xue, Jihui Li, Shujun Chen, Fanli Meng, Xiangjin Chen, Lin Zhao

**Affiliations:** 1 State Key Laboratory of Smart Farm Technologies and Systems, Northeast Agricultural University, Harbin, China; 2 Jilin Academy of Agricultural Sciences, China Agricultural Science and Technology Northeast Innovation Center, Changchun, China; 3 Institute of Soybean Research, Heilongjiang Provincial Academy of Agricultural Sciences, Harbin, China; 4 Key Laboratory of Soybean Molecular Design Breeding, State Key Laboratory of Black Soils Conservation and Utilisation, Northeast Institute of Geography and Agroecology, Chinese Academy of Sciences, Harbin, China; 5 Institute of Soybean Breeding, Heihe Branch of Heilongjiang Academy of Agricultural Sciences, Heihe, China

**Keywords:** *GmMYB176*, *GmSAUR*, isoflavone, secondary metabolism, soybean

## Abstract

**Introduction:**

Isoflavones possess significant health benefits, including anti-inflammatory, anti-cancer, and antioxidant properties. Soybean (*Glycine max* (L.) Merr) is an effective natural source of isoflavones for humans. Thus, it is important to breed soybean varieties with enhanced isoflavone content and to elucidate the secondary metabolic pathways involved. Small auxin-up RNA (SAUR) genes constitute the largest family responsive to auxin, however, few studies have addressed their roles in soybean flavonoid metabolism.

**Methods:**

In this study, flavonoid-targeted metabolites were measured in *GmSAUR:GmSAUR* transgenic soybeans. Chromatin immunoprecipitation sequencing (ChIP-seq) was performed to identify potential target genes regulated by GmSAUR. Additionally, RNA‑Seq was performed to identify downstream genes regulated by 35S:*GmMYB176*, including *GmC4H, GmIF7MaT, GmCYP450 84A1‑like*, and *GmCYP450 84A1*.

**Results:**

The transgenic soybeans exhibited higher contents of daidzin and genistin compared to controls. ChIP-seq revealed that GmSAUR binds to the promoter region of *GmMYB176*, thereby promoting the expression of downstream genes including cinnamate 4-hydroxylase (*GmC4H*), isoflavone 7-O-beta-glucoside 6′′-O-malonyltransferase (*GmIF7MaT*), cytochrome P450 84A1-like (*GmCYP450 84A1-like*), and cytochrome P450 84A1 (*GmCYP450 84A1*). This regulatory cascade ultimately led to increased accumulation of soybean isoflavones.

**Discussion:**

These findings indicate that the *GmSAUR* gene facilitates soybean isoflavone biosynthesis by modulating the expression of *GmMYB176*, providing new insights into the genetic improvement of isoflavone content in soybean.

## 1 Introduction

Isoflavones are flavonoids, belong to the group of secondary metabolites. As weak phytoestrogens, isoflavones have the functions of phytoalexins in plants. When plants are under stress, they can synthesize and accumulate low-molecular-weight compounds (Dakora and Phillips, 1996). Furthermore, within plant protective mechanisms, isoflavones act as stress-relieving intermediaries with antioxidant effects, aiding in the quelling of reactive oxygen radicals produced during stressful circumstances (Kim, 2021). Isoflavones can also promote rhizobia growth and reproduction, nodule development, and nitrogen fixation ability, which is important for the nodulation of soybean roots (Hong et al., 2006; Subramanian et al., 2006). Isoflavones are crucial for both plant development and resilience against harsh conditions, as well as for human health. Soybeans are rich in high-quality proteins and a variety of beneficial physiologically active substances and are an effective source of natural isoflavones in humans. Primarily found in soybeans, the three key isoflavones are genistin, daidzin, and glycitin. Genistin, a key isoflavone compound found in high levels in soybeans, has been shown to potentially help prevent and treat cancer and certain long-term inflammatory diseases. Notably, genistin inhibits tumor cell angiogenesis and metastasis, promotes apoptosis, induces cell cycle arrest, and regulates intracellular signaling pathways to prevent the development of cancer (Nagaraju et al., 2013; Spagnuolo et al., 2015). The primary source of isoflavones in plants is a unique branch of the phenylpropanoid metabolic pathway, which necessitates the catalysis of several enzymes. The two most significant of them are isoflavone synthase (IFS) and chalcone synthase (CHS). Several transcription factors regulate this synthetic pathway, these factors include MYB, bHLH (basic helix-loop-helix), WRKY, WD40 repeat-containing protein and MADS-box. Among these, the MYB protein is considered a key component because it regulates the expression patterns of specific genes (Stracke et al., 2001; Du et al., 2012; Li et al., 2013).

MYB transcription factors constitute a large family of proteins, particularly abundant in eukaryotes. MYB proteins have a highly conserved MYB domain at the N-terminus; this domain generally has four different amino acid repeats (R1, R2, R3, and R4), each approximately 52 to 53 amino acids long (Dubos et al., 2010). Plant MYBs are usually composed of R2 and R3 repeats, and R2R3-MYB TFs account for the largest proportion of all MYB TFs (Romero et al., 1998). However, *GmMYB176* is an R1 MYB TF with an R1 domain that is highly homologous to R1 MYBs from different plant species (Yi et al., 2010). Numerous biological processes, including reactions to hormonal cues, are mediated by MYB transcription factors (Zhang et al., 2024), regulation of the growth and development of plants (Wang et al., 2024a; Wang et al., 2024b), resistance to biotic or abiotic stresses (Liu et al., 2022; Yang et al., 2022), and regulation of plant metabolic pathways (Liu et al., 2015; Battat et al., 2019; Li et al., 2022). MYB176 is essential for regulating flavonoid synthesis, which controls this intricate metabolic process. For example, MYB176 from soybeans (*Glycine max* (L.) Merr; GmMYB176) regulates the expression of CHS8 and affects the synthesis of isoflavones in soybeans by transactivating the CHS8 promoter (Yi et al., 2010). The localization of *GmMYB176* within cells is regulated by the interaction between *SGF14* and *GmMYB176*, which impacts isoflavone production and target gene expression in soybeans (Li et al., 2012). The co-overexpression of *GmMYB176* and *GmbZIP5* activates isoflavone biosynthesis in soybean roots and alters isoflavone levels (Anguraj Vadivel et al., 2021). MicroRNAs (miRNAs) have been shown to mediate the soybean isoflavone biosynthesis pathway. Indeed, one study showed that *MYB176* was a target of *miR-5030* and that there was a perfect negative correlation between *GmMYB176* and *miR-5030* expression (Gupta et al., 2019).

The largest gene family implicated in auxin-related activities is the small auxin-up RNA (*SAUR)* gene family. *SAUR* genes are expressed in soybean hypocotyls induced by exogenous auxin and were found to be present as auxin-induced transcripts (McClure and Guilfoyle, 1987). *SAUR* genes generally lack introns and exist in clusters. Most upstream regions of their promoters contain auxin response elements, which respond quickly to auxin (Li et al., 1994; Hagen and Guilfoyle, 2002; Wu et al., 2012). There is a highly conserved downstream element in the 3′ untranslated region (UTR) of *SAUR* genes, which causes the mRNA encoded by the *SAUR* gene to be unstable, indicating that the *SAUR* gene can be regulated after transcription (Sullivan and Green, 1996). The SAUR protein is highly conserved and has a SAUR-specific domain (SSD) core region composed of approximately 60 amino acids (Park et al., 2007). SAUR proteins are involved in plant growth and development, and *SAUR* genes in the same branch exhibit functional similarity (Zhang et al., 2021). *SAUR* genes have a wide range of effects on plant growth and development regulated by auxin signaling; for example, these genes affect cell elongation by modulating auxin transport in plants (Spartz et al., 2012; Kong et al., 2013; Huang et al., 2023), participate in plant stress (Chen et al., 2014; Qiu et al., 2020), play roles in anthocyanin accumulation in plants, and control leaf senescence (Kant et al., 2009; Hou et al., 2013). Since the *GmSAUR* gene has been reported to be involved in the regulation of soybean stem elongation and gibberellin (Sun et al., 2023), gibberellin mediates the synthesis of many flavonoids (Loreti et al., 2008; Cheng et al., 2015; Tan et al., 2019; Li et al., 2020; Lu et al., 2022), which prompted us to study whether the *GmSAUR* gene involved in GA regulation can affect the synthesis of soybean flavonoids.

In this study, to better understand how the *GmSAUR* gene affects soybean isoflavone content, we integrated chromatin immunoprecipitation-sequencing (ChIP-seq) data and found that soybean *GmSAUR* it binds directly to the promoter region of *GmMYB176*. The overexpression of *GmMYB176* in hairy roots activated downstream genes related to isoflavone synthesis, such as cinnamate 4-hydroxylase (*GmC4H*), isoflavone-7-O-beta-glucoside 6′′-O-malonyltransferase (*GmIF7MaT*), cytochrome P450 84A1-like (*GmCYP450 84A1-like*), and cytochrome P450 84A1 (*GmCYP450 84A1*), further promoting soybean isoflavone accumulation. These results provide a new perspective on the biosynthesis of isoflavones in plant secondary metabolism.

## 2 Materials and methods

### 2.1 Plant materials and growth conditions

All genetic modification experiments used soybean variety DN50 as the wild-type background material, and its seeds originated from Northeast Agricultural University in Harbin, China. Genetically edited transgenic soybean seeds capable of over-expressing *GmSAUR* protein were cultivated in Harbin (45°75′N, 126°63′E) utilizing an 8-hour darkness and 16-hour light photoperiod management system.

*Nicotiana benthamiana* plants were cultured in vermiculite and turfy soil at a ratio of 1:1 at a consistent 25 °C temperature. After *Agrobacterium* infection, the transiently transformed *N. benthamiana* plants were grown under SD (8/16 h light/dark) conditions for 2 days, after which the sample of leaves was taken.

### 2.2 Identification and phylogenetic relationship analysis of *GmSAUR* genes

We used the comprehensive and eclectic ClustalW (https://clustalw.com/) to compare multiple complete amino acid sequences of the GmSAUR protein in soybean (*Glycine max*) with those of other plant species. MEGA7.0 neighbor-joining approach was applied to the aligned sequences in order to create a phylogenetic tree. The tree was subsequently visualized and refined with EvolView, and its robustness was evaluated with 1,000 bootstrap replicates.

### 2.3 Plasmid construction and generation of transgenic soybeans

The recombinant vectors *GmSAUR: GmSAUR-FLAG* and *GmSAUR: GST* were successfully generated using established molecular cloning techniques (Sun et al., 2023). The *GmSAUR: GmSAUR-FLAG* vector was constructed by cloning the *GmSAUR* native promoter and its CDS into *pENTRY-3F6H* via homologous recombination, followed by LR recombination into *pB7WG2-no35S*.

To build the *GmMYB176*:LUC fusion vector, using the genomic DNA of ‘DN50’ as a template and amplified its promoter segment using *GmMYB176*:*LUC-F* and *GmMYB176*:*LUC-R* primers (**Supplementary Table S1**). A homologous recombination method was used to linearize the PCR result with SmaI, and then the pGreenII-0800-LUC vector was neatly spliced into it. The newly constructed fusion plasmid was transformed into *Agrobacterium* GV3101 (pSoup-p19).

To build *35S: GmMYB176-GFP*, we used the cDNA of DN50 variety as a template and used the primer pairs *GmMYB176-TOPO-F* and *GmMYB176-TOPO-R* (**Supplementary Table S1**) to amplify the 858 bp *GmMYB176* coding sequence by PCR, inserted it into the vector pENTR/D-TOPO, and subsequently transferred it into the pGWB506 vector via an LR cloning reaction. *Agrobacterium tumefaciens* strain *EHA105* was then infected with the resultant fusion plasmids.

The *GmMYB176* RNAi construct was generated as described by Yi et al. (2010). and introduced into soybean hairy roots via *A. rhizogenes*-mediated transformation.

### 2.4 Transformation and detection of soybean hairy roots

The soybean hairy root transformation procedure was carried out according to the previously established protocol (Xu et al., 2023). Assessment of *GmMYB176* gene expression in transgenic cotyledon hairy root-derived plants involved GFP fluorescence and RT-qPCR analysis.

### 2.5 RNA-seq, statistical analysis, and RT-qPCR analysis

Trifoliate leaves were collected from 23-day-old wild-type (WT) and *GmSAUR* soybean plants at ZT 12h under long-day (LD) conditions for RNA-seq analysis as described previously (Yamaguchi et al., 2014). Total RNA was isolated from soybean plants using TRIzol reagent, as previously described (Zhao et al., 2013). The extracted RNA was reverse-transcribed into cDNA, which served as the template for quantitative polymerase chain reaction (RT-qPCR) assays conducted on an ABI Prism 7500 sequence detection system (Applied Biosystems, Foster City, CA, USA). *GmActin4* was employed as an endogenous reference gene for normalization. Three distinct biological replicates were used in each experiment, along with three technical replicates. See **Supplementary Table 1** for the primer sequences used in RT-qPCR.

### 2.6 ChIP-seq and ChIP-qPCR

Approximately 1 g of trifoliate leaves was collected from both WT and transgenic plants at ZT 12h, followed by immediate fixation and quenching with glycine, as previously described (Sun et al., 2023). We used a mouse-derived monoclonal anti-FLAG antibody (anti-FLAG M2, item No. F1804;Sigma-Aldrich, St. Louis, MO, USA) to conduct chromatin immunoprecipitation experiments. Capture antibody-chromatin complexes were incubated with Protein G magnetic beads (Invitrogen). The primer sequences utilized in this experiment are listed in **Supplementary Table S1**, with *GmActin4* serving as the internal reference gene.

### 2.7 EMSA

A direct interaction between the *GmSAUR* protein and the *GmMYB176* promoter regions was tested by (EMSA). Oligonucleotide probes encompassing the candidate binding sites within the promoter were synthesized and subjected to 5′-end labeling with biotin. Probe sequences are shown in **Supplementary Table S1**. We performed this analysis using a previously described method (Sun et al., 2023).

### 2.8 Transient transcription dual-luciferase assay

To measure the effects of *GmSAUR* on the promoter of *GmMYB176*, the recombinant constructs *GmSAUR: GmSAUR*-3F6H-pB7WG2 (Sun et al., 2023) and *GmMYB176*:*LUC* were co-expressed in *N. benthamiana* leaves via agroinfiltration. Following infiltration, plants were cultivated for 48 hours under controlled conditions. Prior to imaging, leaves were sprayed with a solution of d-luciferin potassium salt. Luminescent signals were captured using a low-light imaging system. In addition, we also collected leaf samples and used the dual luciferase reporter gene detection kit for quantitative analysis in strict accordance with the manufacturer’s operating instructions.

### 2.9 Subcellular localization in *N. benthamiana* mesophyll cells

To visualize nuclear localization, a red fluorescent protein (RFP)-tagged histone H2B construct (H2B-RFP) was employed as a nuclear marker (Goodin et al., 2002). 48 hours after transfection, we observed the leaf samples with the help of a laser confocal microscope for image acquisition.

The *35S:GmMYB176-GFP* plasmid was first added into *A. tumefaciens* strain *EHA105*. Subsequently, the recombinant bacteria were used to transform *N. benthamiana* (Hu et al., 2013). To visualize nuclear localization, a red fluorescent protein (RFP)-tagged histone H2B construct (H2B-RFP) was employed as a nuclear marker (Goodin et al., 2002). Leaves were examined using a microscope equipped with confocal laser scanning to obtain images 48 hours after transfection.

### 2.10 Extraction and determination of metabolites

Three biological duplicates of each sample were created for metabolomic studies. The experiment used a SCIEX QTRAP6500 + mass spectrometer, which was connected to an ultra-performance liquid chromatography-mass spectrometry system. This device has an IonDrive Turbo V ESI ion source, and each injection volume is 2 μL. The mobile phase is a mixture of 0.1% formic acid aqueous solution and acetonitrile. Beijing Biomarker technology Company helped us with the extraction and testing.

## 3 Results

### 3.1 Sequence analysis of *GmSAUR*

**Figure 1:**
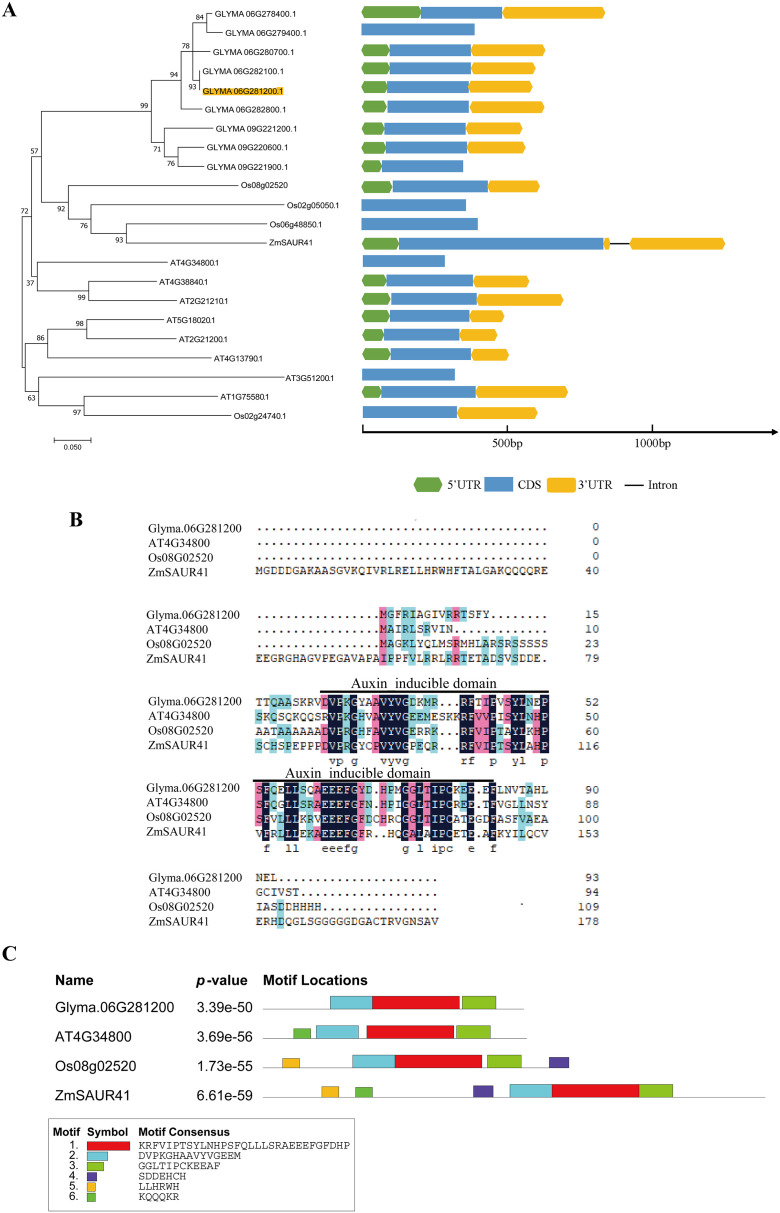
Sequence analysis of the *GmSAUR*. **(A)** Phylogenetic tree analysis was conducted on the protein sequences of GmSAUR and other species with high similarity, sourced from the NCBI database. Four species were included in the phylogenetic tree construction: Several plant species serve as common models for this research. These include soybean (*Glycine max*), Arabidopsis (*Arabiana thaliana*), rice (*Oryza sativa*), and maize (*Zea mays*). These amino acid sequences were obtained by referring to data from the Phytozome database. MEGA 7.0 software was used to conduct phylogenetic analysis and present the branch lengths of the evolutionary tree on a scale of 0.05 to show the degree of differentiation between sequences. **(B)** The GmSAUR protein from soybean was analyzed through multiple sequence alignment with similar proteins in *Arabidopsis thaliana* (*At*), *Oryza sativa* (*Os*), *Zea mays* (*Zm*). Fragments of these protein sequences that respond to auxin have been underlined. **(C)** The MEME software was utilized to analyze the distribution of conserved motifs within the GmSAUR protein and other proteins with high sequence homology, identifying six distinct motifs. The sequences of these six motifs are presented below.

The leaves of “Dongnong 50” (DN50) were used to clone the full-length cDNA sequence of *GmSAUR* (Glyma.06G281200) using the polymerase chain reaction (PCR). The cDNA sequence of *GmSAUR* was 599 bp, containing a 92-bp 5′ UTR, a 225-bp 3′ UTR, and a 282-bp open reading frame, encoding 93 amino acids. SAUR protein is highly conserved and has an SSD core region, which is composed of approximately 60 amino acids (Park et al., 2007). In order to investigate the evolutionary connections among soybean *GmSAUR* genes, the SAUR protein sequences from four species—*Glycine max*, *Arabidopsis thaliana*, *Zea mays*, and *Oryza sativa*—were selected for alignment. We used MEGA7 to make a phytogenetic tree, and the results showed that genes in close proximity in the sequence may have functional connections (**Figure 1A**). Multiple sequence alignments of GmSAUR, AT4G34800, Os08G02520, and ZmSAUR41 showed that the GmSAUR protein contained a conserved auxin-inducible domain (**Figure 1B**). Additionally, analysis of the conserved motif distribution of all SAUR proteins showed that all proteins had motifs 1, 2, and 3 (**Figure 1C**).

### 3.2 Phenotypic analysis of isoflavone content in *GmSAUR* soybean seeds

**Figure 2:**
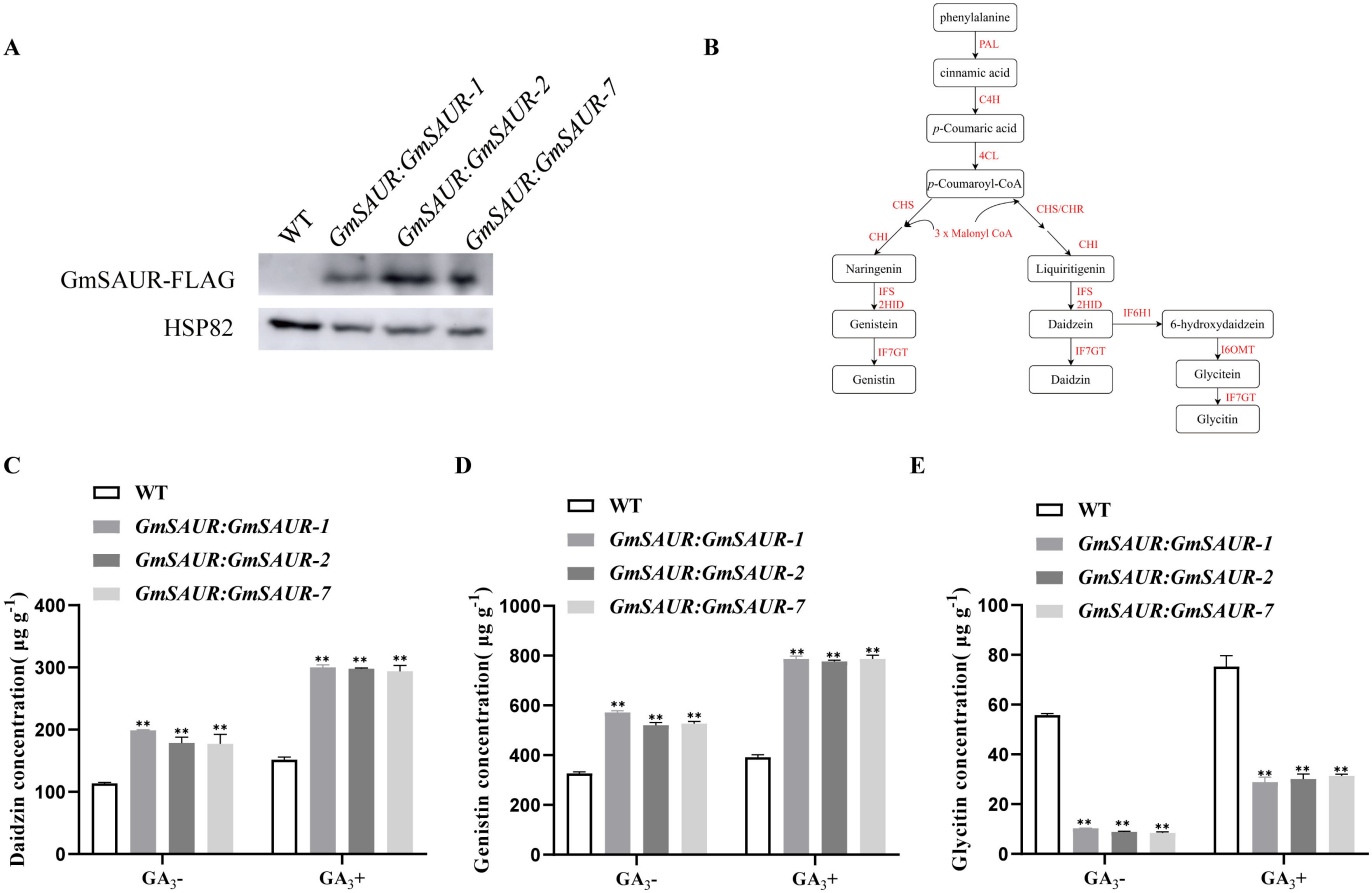
Phenotypic analysis of isoflavone content in *GmSAUR* soybean seeds. **(A)** The seeds of T_4_ GmSAUR: GmSAUR and the wild type (WT) were analyzed using immunoblotting with an anti-FLAG antibody, with soybean HSP82 serving as an internal reference. **(B)** The soybean isoflavone synthesis pathway. The red font represents the catalyzed enzyme, and the black font represents the synthesized substance. **(C–E)** The content of main isoflavones components in *GmSAUR: GmSAUR* soybean seeds treated with MS medium containing 0 and 10 μM GA_3_. Values are presented as means ± standard deviation (SD). The experiment used biological samples and each group was repeated three times. Symbols on the figure mean significant differences compared to the control group. One-way ANOVA for statistical analysis was used. **p < 0.01.

In the three T_4_ transgenic soybean lines, *GmSAUR: GmSAUR-1*, *GmSAUR: GmSAUR-2*, and *GmSAUR: GmSAUR-7*, seeds at the R7 stage were detected using western blotting, and the GmSAUR-FLAG protein (23 kDa) was detected (**Figure 2A**). Additionally, compared with the wild type, the mRNA transcription level of *GmSAUR* in *GmSAUR: GmSAUR* lines was increased, and the transcriptional abundance was promoted by GA_3_ (Sun et al., 2023). (**Supplementary Figure 1A**). In the synthesis pathway of soybean isoflavones, various catalytic enzymes and key enzyme genes regulate various reactions on the synthesis pathway (Bennett et al., 2004). Isoflavones also belong to phenylpropanoid skeleton compounds, in which aglycone compounds (Daidzein, Genistein and Glycitein) are directly synthesized by phenylalanine metabolic pathway, and the remaining Glycosides, Acetylglycosides and Malonylglycosides are synthesized by glycosylation or acylation of aglycone compounds (Funaki et al., 2015; Fujino et al., 2018; Dong and Lin, 2021). The soybean isoflavone synthesis pathway is shown in **Figure 2B**. In order to test whether the GA_3_-promoted *GmSAUR* gene will affect the production of isoflavones in soybean seeds, we measured the concentration of isoflavones (Daidzin, Genistin, Glycitin) in transgenic *GmSAUR: GmSAUR* soybean under GA_3_ treatment. The findings indicated that the concentration of daidzin and genistin in GA_3_-promoted *GmSAUR: GmSAUR* transgenic soybean were substantially greater than those found in the wild-type (WT) soybeans, conversely, the concentration of glycitin was lower than that of the WT soybeans (**Figures 2C–E**).

### 3.3 *GmSAUR* directly promoted the expression of *GmMYB176* in soybean

**Figure 3:**
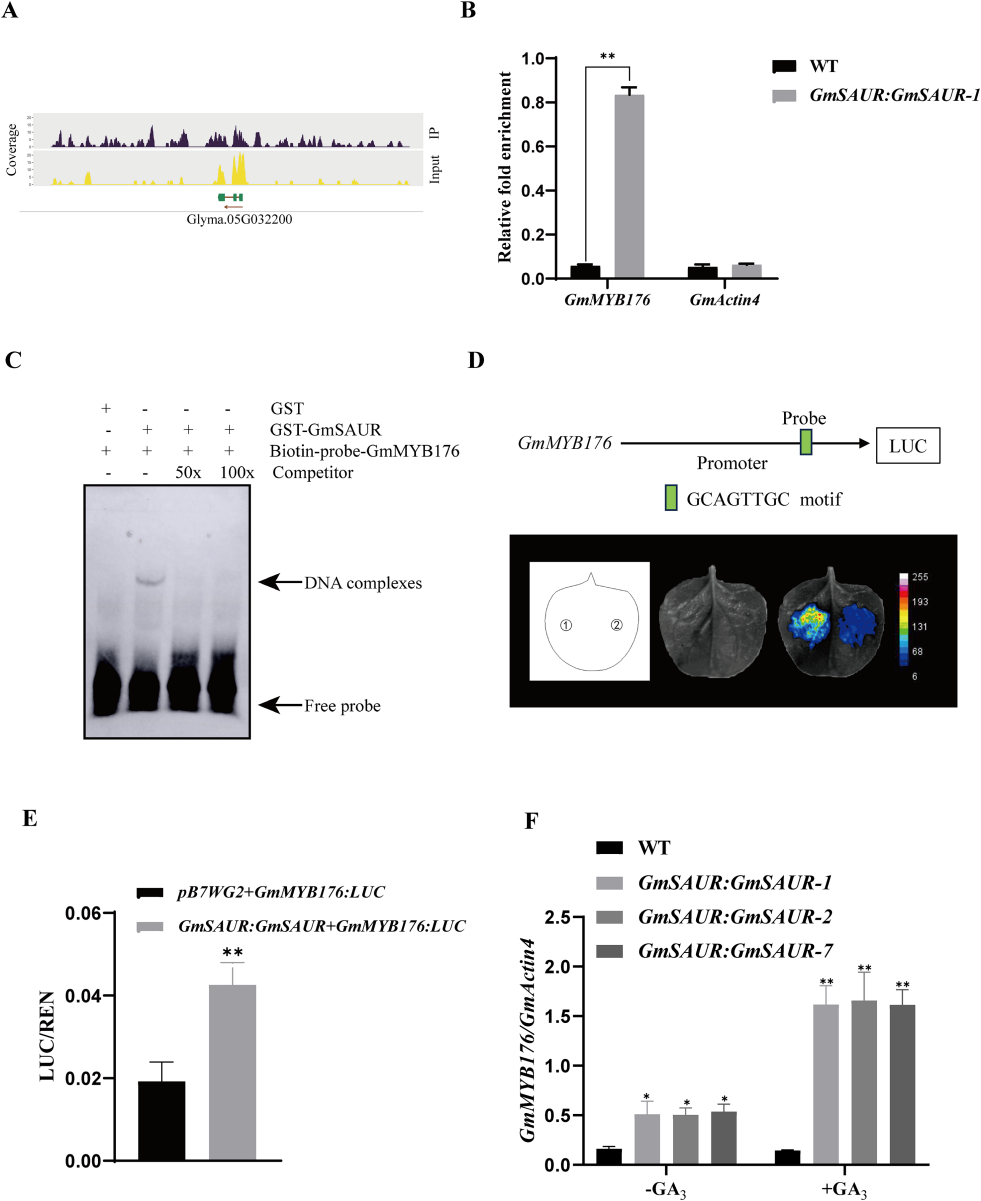
Identify the target genes of GmSAUR and the binding of GmSAUR to the *GmMYB176* promoter throughout the soybean genome. **(A)** ChIP-seq data visualization results, showing the original read peak map of specific gene loci through the Integrated Genomics Viewer. The transcription direction is indicated by the arrow, the bars indicate the transcripts of the gene. **(B)** ChIP-qPCR results verified the binding of GmSAUR to the *GmMYB176* promoter region in living cells. Plant growth conditions were consistent with ChIP-seq experiments. The asterisk marks the statistically significant differences between the wild type (WT) and the *GmSAUR: GmSAUR*. The *GmActin4* area served as a negative control. The averages, along with their range of fluctuations, are shown as mean ± SD, calculated from three independent biological replicates, and statistical significance was tested by One-way ANOVA, with **p < 0.01. **(C)** EMSA assays showing that GmSAUR binding to the *GmMYB176* DNA fragments. Unlabeled DNA fragments were added (50-fold and 100-fold molar excess of labeled probes) and used as competitors. GST served as a negative control. **(D)** Luciferase activity of *GmMYB176:LUC* and *GmSAUR: GmSAUR* effector constructs at 12 h after dawn in SDs. 1: *GmSAUR: GmSAUR* + *GmMYB176:LUC*; 2: *pB7WG2* + *GmMYB176:LUC*. Upper panel: physical locations of fragments harboring putative motifs are shown in the schematic diagram. Three independent transfection experiments were performed. **(E)** The LUC/REN relative expression activity of *pB7WG2* + *GmMYB176:LUC* and *GmSAUR: GmSAUR* + *GmMYB176:LUC* in *N. benthamiana* at 12 h after dawn in SDs. An asterisk indicates important changes compared to the control group. The mean ± SD of three biological replicates is used to calculate the values. Student’s t-test(two-tailed) was used to establish statistical significance (**p < 0.01). **(F)** Expression of GmMYB176 in seeds from *GmSAUR: GmSAUR* plants treated with MS medium containing 0 and 10mM GA_3_. Three biological replicates were used for the experiment. A statistically significant difference from the control group is indicated by an asterisk. One-way ANOVA was used to determine statistical significance (**p < 0.01).

Based on our previous genome-wide analysis of ChIP-seq data for *GmSAUR* (Sun et al., 2023), the *GmMYB176* gene, which regulates soybean isoflavone content, was selected for further analysis. The peak sequence was found upstream of the *GmMYB176* transcription start point, according to ChIP-seq data. (**Figure 3A**).

ChIP-qPCR was carried out on GmSAUR: GmSAUR-1 seeds at ZT 12 h under SD conditions in order to confirm putative GmSAUR-binding sites. ChIP-qPCR was used to confirm the ChIP fragment analysis’s finding that the *GmMYB176* promoter region had substantially enriched GmSAUR-binding sites. (**Figure 3B**). *In vitro* binding research was performed using the electrophoretic mobility shift assay (EMSA) to ascertain if GmSAUR is bound directly to the promoter region of *GmMYB176*. GST-GmSAUR significantly reduced the mobility of the probe, indicating that GmSAUR could directly bind to the promoter sequence of *GmMYB176* (**Figure 3C**). A reporter was then constructed using *LUC* driven by the *GmMYB176* promoter. The *GmSAUR: GmSAUR* effector was co-expressed with the *GmMYB176*:*LUC* reporter (**Figure 3D**), and LUC activity was extremely high in correlation with GmSAUR protein expression, indicating that GmSAUR may stimulate *GmMYB176* expression (**Figure 3E**). Additionally, the *GmMYB176* promoter was bound by GmSAUR, and the expression of *GmMYB176* was promoted in the seeds of *GmSAUR: GmSAUR* plants whether or not treated with GA_3_ (**Figure 3F**). Based on this information, it appears that the GmSAUR protein binds to the promoter of *GmMYB176* to increase its activity.

### 3.4 Target metabolome analysis in *GmSAUR* transgenic soybeans

**Figure 4:**
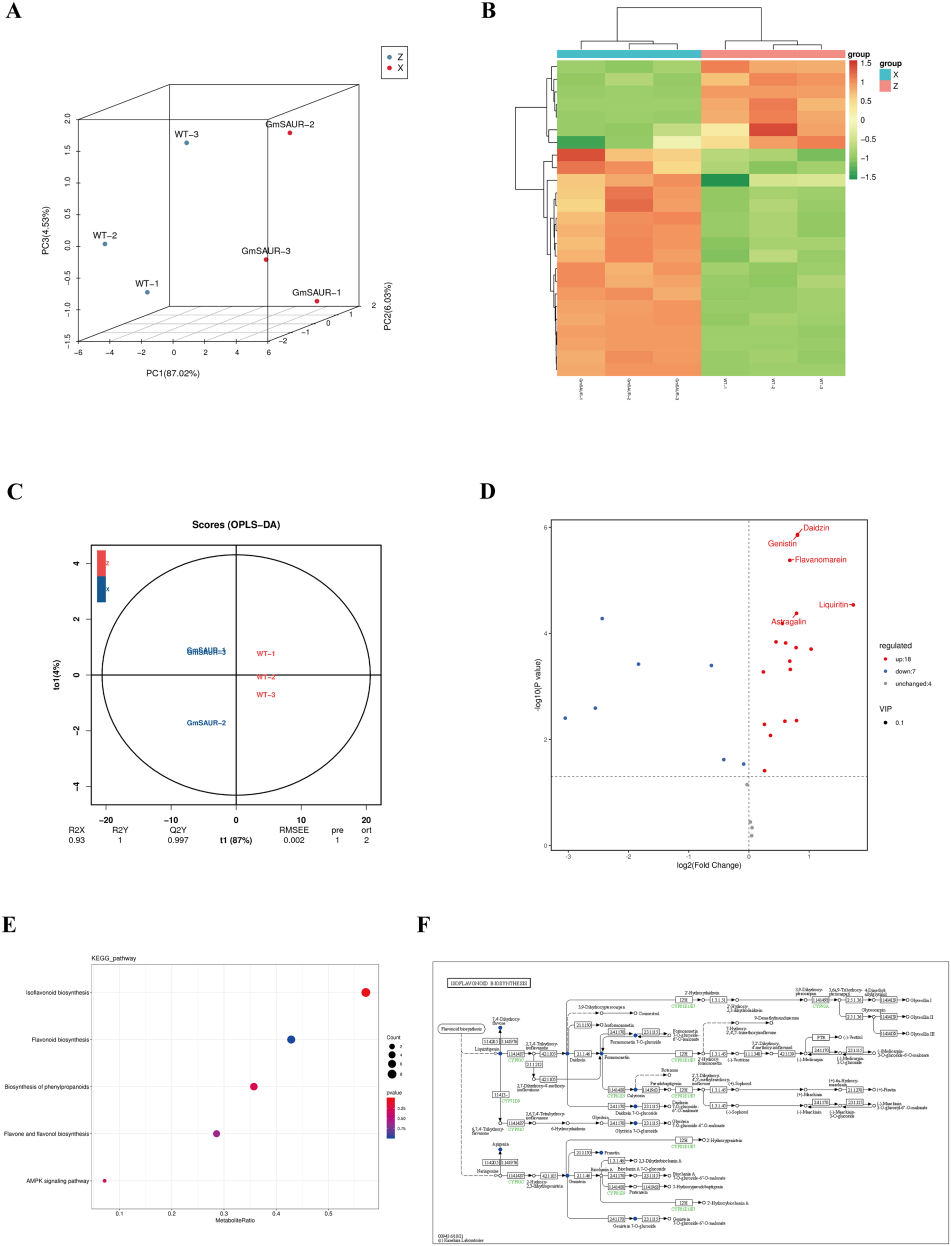
The metabolites analysis in soybean *GmSAUR* seeds. **(A)** Score plot of PCA of metabolite datasets. Each point corresponds to one metabolite profiling experiment. The symbol “Z” indicates wild-type samples. The symbol “x” represents *GmSAUR: GmSAUR* samples. The percentage of coordinate axis represents the contribution of the principal component to the sample difference. **(B)** Heat map visualization of metabolites. Each row represents one metabolite. The y-axis is the quantitative value normalized by the Z-score of the metabolites after hierarchical clustering(color key scale right of the heat map). **(C)** OPLS-DA score plot. The axis ( t1 ) represents the prediction component ( inter-group difference component ),the y-axis ( t2 ) represents the orthogonal component ( intra-group difference component ), and the percentage of the horizontal y-axis represents the proportion of the component in the total variance. **(D)** Volcano plot of flavonoid expression in WT versus *GmSAUR: GmSAUR*. Red symbols denote metabolites that are significantly increased, blue symbols signify those that are significantly decreased, and gray symbols represent metabolites with no significant alteration. **(E)** KEGG enrichment map of *GmSAUR: GmSAUR* and WT differential metabolites. The abscissa (x-axis) shows the enrichment ratio. The ordinate (y-axis) lists the name of the pathway. The color depth of the point represents logP value, and the redder the more significant the enrichment. The size of the point represents the number of differential metabolites enriched. **(F)** Isoflavonoid biosynthesis functional annotation pathway diagram of differential metabolites. Red indicated that the metabolite content was significantly up-regulated, and green indicated that the metabolite content was significantly down-regulated.

To understand how the *GmSAUR* gene affected the metabolic composition of soybean seeds and flavonoid biosynthesis, we performed flavonoid-targeted metabolomic sequencing using *GmSAUR* transgenic soybeans, the concentrations of 200 flavonoids were determined. The contents of other metabolites were shown in **Supplementary Data S1**. Most metabolites did not change significantly, and some metabolites showed different trends in different biological replicates. Although a few metabolites changed, the magnitude was much smaller than that of isoflavones. Principal component analysis showed that the two groups of samples were completely separated along the direction of PC1 (87.02%). The WT group was located in the negative half axis of PC1, and the GmSAUR group was located in the positive half axis of PC1 (**Figure 4A**). The Q2Y value in the OPLS-DA analysis results was greater than 0.9, which also indicated that the model was reliable and could be used to screen differential metabolites (**Figure 4C**). Using the similarity of metabolites to classify the samples, we could more intuitively observe the upregulated or downregulated metabolites in *GmSAUR: GmSAUR* seeds compared with WT seeds (**Figure 4B**; red: high abundance, green: low abundance). These differences were visualized using volcano maps. The point above the significance threshold (dotted line) represents a flavonoid with a significant difference between the two groups, with a log2 (fold change) value greater than 0. The findings showed that the flavonoid content in *GmSAUR* seeds was upregulated compared with that in the WT (**Figure 4D**). The metabolic pathways that were highly enriched among the metabolites that were differentially expressed in *GmSAUR* seeds were annotated using the KEGG database. As shown in **Figure 4E**, differentially expressed metabolites are involved in pathways such as “isoflavonoid biosynthesis,” “flavonoid biosynthesis,” “biosynthesis of phenylpropanoids,” “flavone and flavonol biosynthesis,” and “AMPK signaling pathway.” Among these pathways, we selected the significantly enriched pathway of “isoflavonoid biosynthesis” to visualize the isoflavonoid biosynthesis pathway (**Figure 4F**), highlighting the differential metabolites found in the comparison of WT and *GmSAUR: GmSAUR* seeds. As shown in **Figures 4D, F**, the accumulation pattern of isoflavone metabolites is consistent with the up-regulated expression pattern of the key gene *GmMYB176* in the above isoflavone biosynthesis pathway (**Figure 3F**), which jointly confirms the activation of the pathway. Elucidation of this pathway is crucial for understanding the mechanisms of soybean isoflavonoid production in *GmSAUR* transgenic soybean plants.

### 3.5 Overexpression of *GmMYB176* modulates gene expression during biological processes

**Figure 5:**
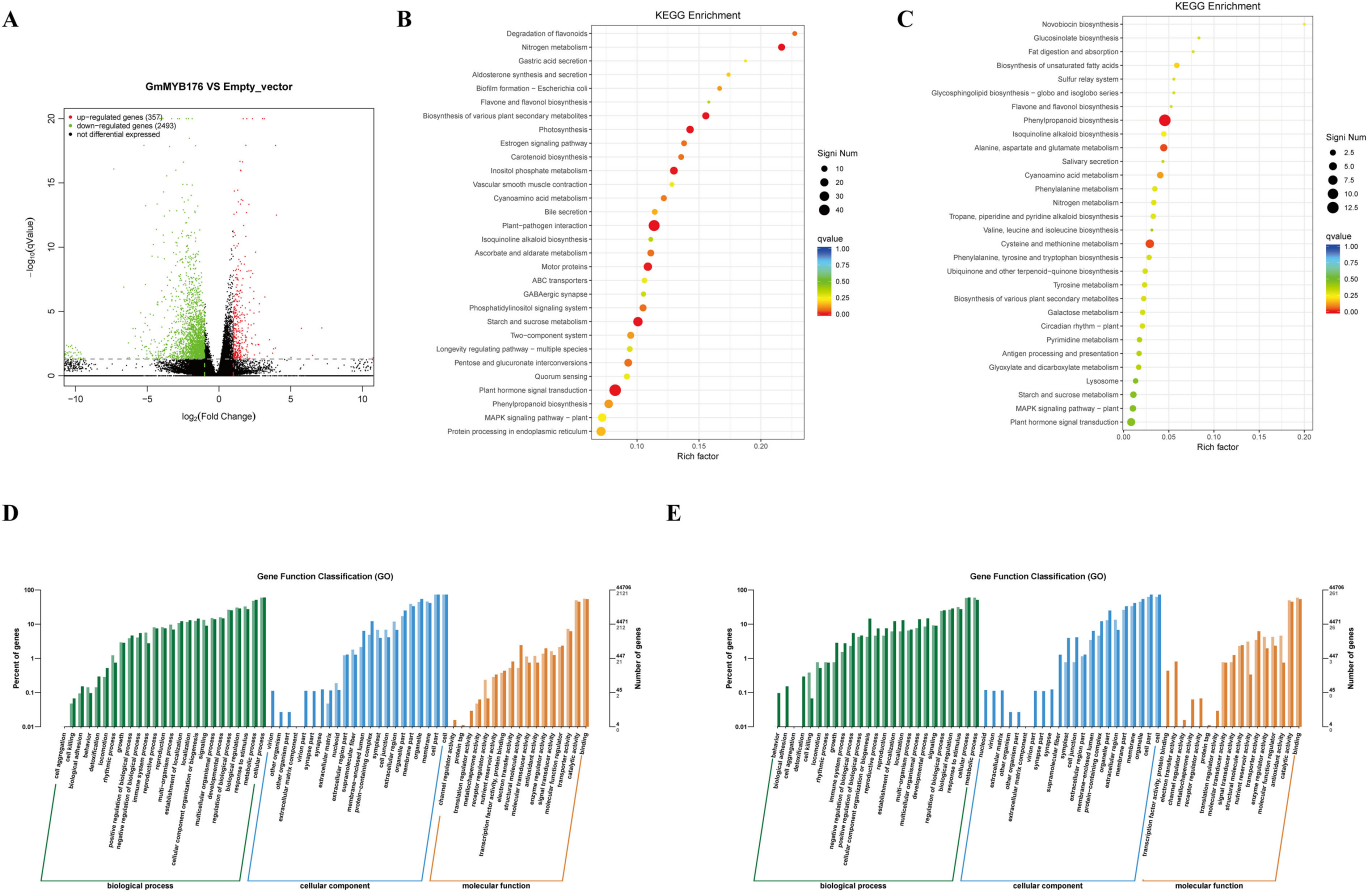
RNA-seq analysis of *35S:GmMYB176* **(A)** Differentially expressed gene scatter plot. Each pointsymbolizes a gene: red denotes overexpression, green signifies underexpression, and black denotes no change in expression. **(B)** KEGG annotation for genes with decreased differential expression. **(C)** KEGG annotation for genes with increased differential expression. **(D)** GO annotation for down-regulated differential genes. The numerical light color on the bar chart and the ordinate axis represents the difference basis. **(E)** GO annotation for up-regulated differential genes.

To further elucidate the molecular network regulated by *GmMYB176*, we employed RNA-seq to compare the overall expression profiles of soybean genes in empty vector and *35S:GmMYB176* hairy roots. Each sample yielded approximately 57.94 million clean RNA-seq reads, with 94.42% mapping to the current soybean reference genome. Differential expression was determined using a *p*-value of less than 0.05 and a |fold change| greater than 2. There were found to be 2850 genes with differential expression, 357 of which were upregulated and 2493 of which were downregulated (**Figure 5A**).

The biological roles of these differentially expressed genes were then categorized and annotated using the KEGG database. As depicted in **Figures 5D, E**, the differentially expressed genes in *GmMYB176* transgenic soybeans, compared to those in the empty vector control, were primarily involved in phenylpropanoid biosynthesis and various metabolic pathways. Subsequently, Gene ontology (GO) enrichment analysis was performed on the differentially expressed genes to investigate the potential biological functions of *GmMYB176*, based on the primary biological functions of these genes. The GO enrichment analysis for cell component functions (**Figures 5B, C**) revealed that cells, membranes, and organelles were the primary locations for both increased and decreased genes. In the category of biological processes, those genes whose expression changes are mainly involved in metabolic processes, cellular processes., and the regulation of biological processes. Among those genes with particularly large changes in expression, their main functions can be divided into three categories at the molecular level: the ability to bind with other molecules, the ability to have enzymes or catalysis, and the role of transportation.

### 3.6 35S:*GmMYB176* affected the expression of key isoflavone synthase genes

**Figure 6:**
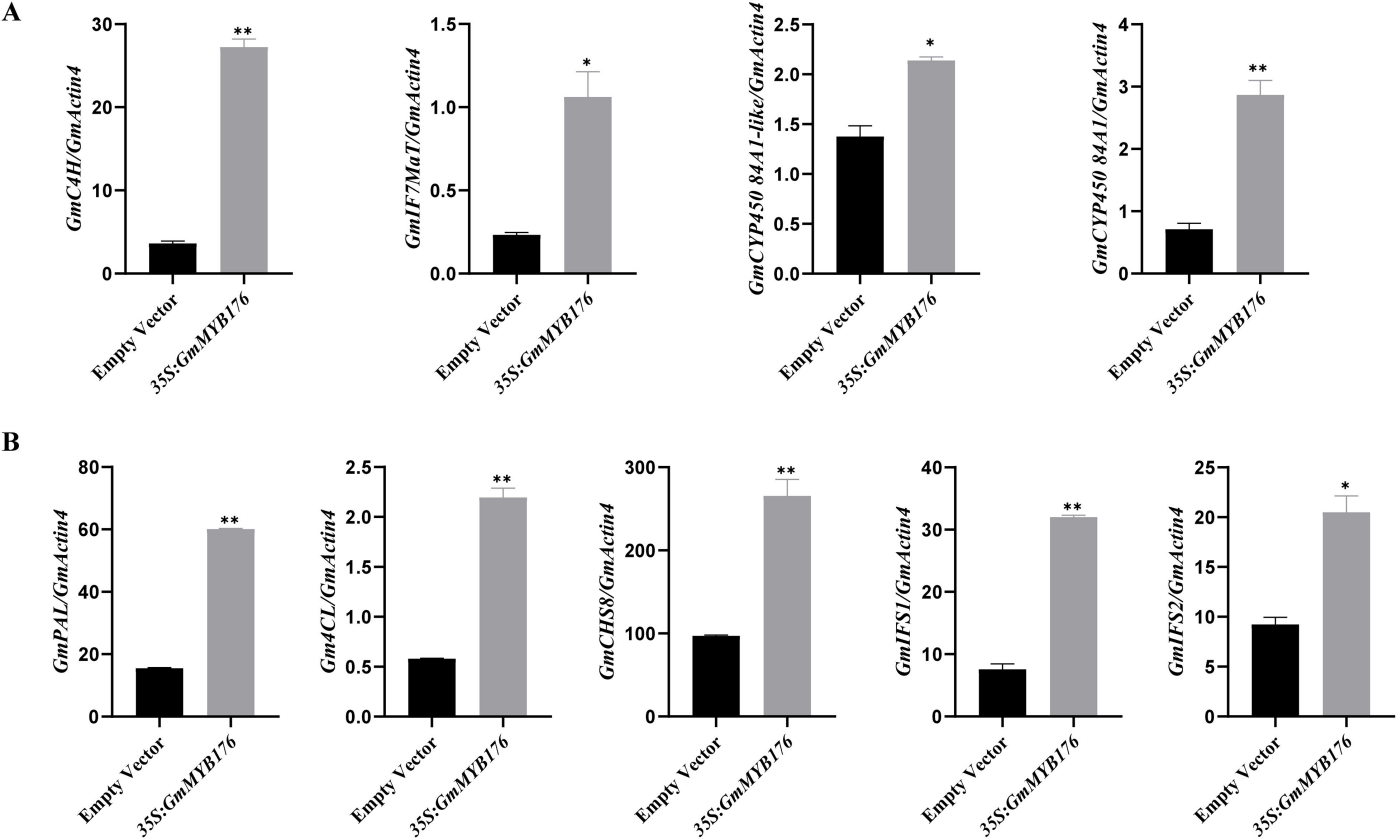
RT-qPCR was employed to assess the expression of isoflavone synthesis genes in the *35S:GmMYB176* construct and the control vector. **(A)** The relative expression levels of *GmC4H, GmIF7MaT, GmCYP450 84A1-li*ke, and *GmCYP450 84A1 in 35S:GmMYB176* and empty vector plants, respectively. **(B)** The relative expression levels of *GmPAL*, *Gm4CL, GmCHS8, Gm*IFS1, and *GmIFS2 in 35S:GmMYB176* and empty vector plants, respectively. Each experiment included three technical replicates. The data were displayed as mean ± standard deviation (SD). These values were calculated based on the results of three independent replicates of experiments. Asterisks indicate significant differences between *35S:GmMYB176*-transgenic plants and empty vector controls (*P < 0.05, **P < 0.01; Two-tailed Student’s t-test).

Functional study of the genes with differential expression in plants that overexpress *GmMYB176* revealed that four genes related to isoflavone synthesis were upregulated: *C4H*, *IF7MaT*, cytochrome P450 84A1-like, and cytochrome P450 84A1. *IF7Ma*T was first characterized in chickpea, it is a member of the flavonoid glycoside-specific acyltransferase in the BAHD family and is mainly expressed in roots (Koester et al., 1984; Suzuki et al., 2007). Cytochrome P450 monooxygenases (CYP450s) catalyze NADPH-dependent substrate hydroxylation and are involved in flavonoid biosynthetic pathways (Montellano, 2005). Notably, RT-qPCR showed that the relative mRNA expression levels of *GmC4H*, *GmIF7MaT*, *GmCYP450 84A1-like*, and *GmCYP450 84A1* in *35S*:*GmMYB176* hairy roots were higher than those in roots transfected with the empty vector (**Figure 6A**). The mRNA expression levels of other isoflavone biosynthesis-related enzyme genes, namely *GmPAL*, *Gm4CL*, *GmCHS8*, *GmIFS1*, and *GmIFS2*, were also higher in *35S*:*GmMYB176* hairy roots than in those transfected with the empty vector (**Figure 6B**). The expression levels of these genes were also significantly higher in *GmSAUR* overexpression lines than in WT (**Supplementary Figure S3**). These findings raise the possibility that *GmSAUR* and *GmMYB176* regulates soybean isoflavones via upregulating the expression of genes that encode isoflavone synthesis-related enzymes.

To functionally validate the role of *GmMYB176* in isoflavone accumulation, we generated *GmMYB176* RNAi hairy roots following the method described by Yi et al. (2010). Quantitative RT-qPCR confirmed that *GmMYB176* transcript levels were significantly reduced in RNAi lines compared with empty vector controls (**Supplementary Figure S1**). Isoflavone content analysis revealed that total isoflavone levels were markedly decreased in *GmMYB176*-silenced hairy roots relative to controls (**Supplementary Figure S1**), consistent with previous findings (Yi et al., 2010). These results demonstrate that *GmMYB176* positively regulates isoflavone accumulation in soybean hairy roots.

**Figure 7:**
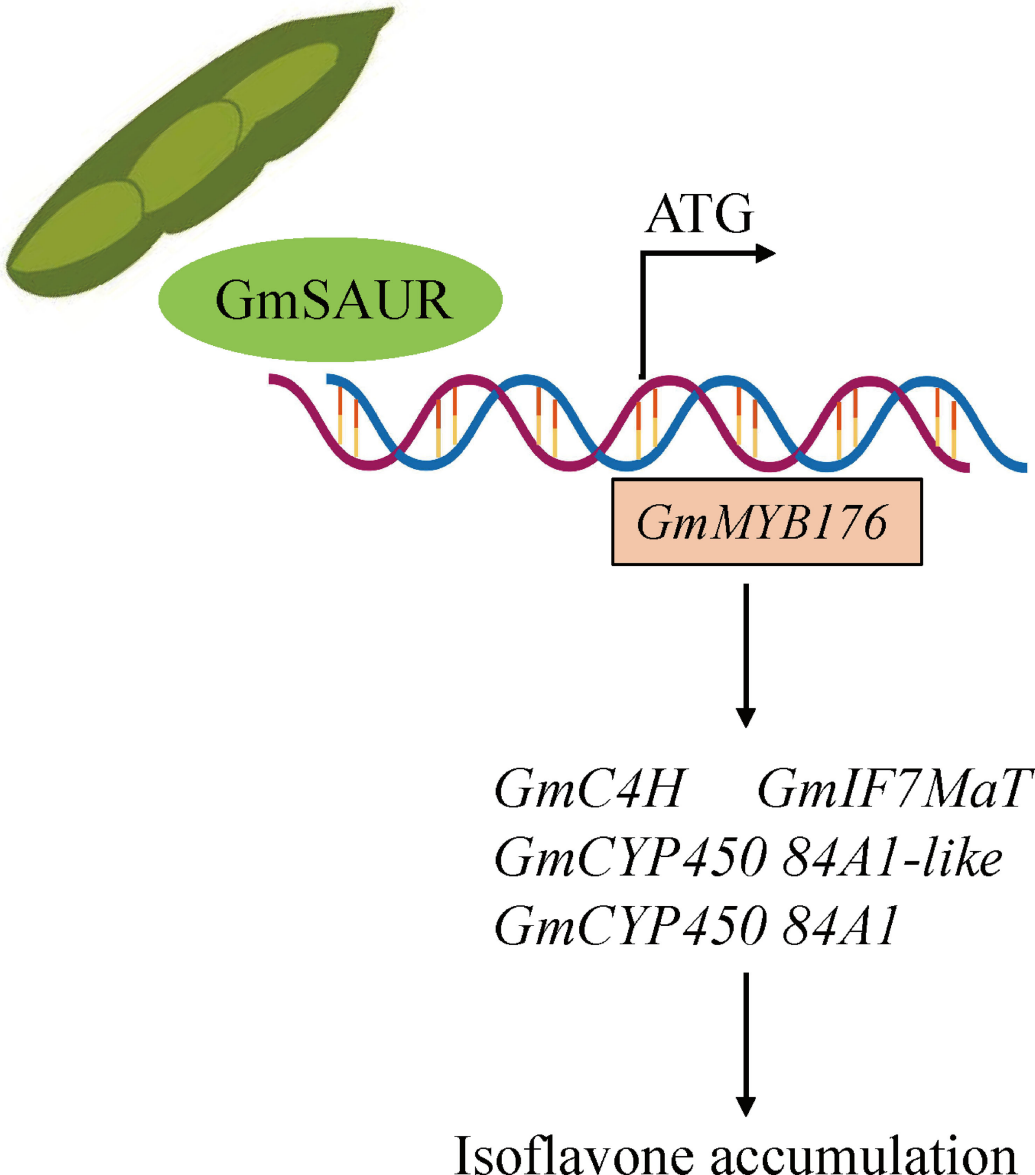
Proposed is a functional model of *GmSAUR* involved in the regulation of soybean isoflavone content. The solid arrow signifies the activation of transcription.

We propose a model for the role of GmSAUR in controlling the isoflavone content of soybeans based on the previously mentioned experimental findings (**Figure 7**). In this model, GmSAUR directly binds to the promoter of *GmMYB176*; increases the transcriptional abundance of genes such as *GmC4H*, *GmIF7MaT*, *GmCYP450 84A1-like*, and *GmCYP450 84A1*; and promotes the accumulation of soybean isoflavones.

## 4 Discussion

Flavonoids represent a large class of polyphenols in terrestrial plants. The accumulation of these secondary metabolites, especially flavones and anthocyanins, enables plants to respond to various biotic and abiotic factors in the environment (Ma et al., 2014; Li et al., 2017; Qin et al., 2021; Jiang et al., 2025). Flavonoids are an important medium for gibberellin to regulate various biological processes. The biosynthesis of flavonols regulated by gibberellin is necessary for the growth of main roots in response to environmental signals. In the presence of gibberellin, DELLA can be degraded, reducing the transcriptional activity of MYBs, the lower flavonoid content allows more auxin to be transported to root tip cells, promoting cell division and root growth of Arabidopsis thaliana (Tan et al., 2019). In gibberellin-treated rice seedlings, the flavonoid accumulation level in the root tip of *ks1* mutant was significantly increased and negatively correlated with gibberellin content (Li et al., 2020). Exogenous gibberellin treatment of grape (Vitis labrusca × Vitis vinifera) significantly up-regulated the expression levels of *CHS*, *F3H* and *PAL* involved in flavonoid biosynthesis (Cheng et al., 2015). The increase of gibberellin promoted the expression of *GbCHS* in Ginkgo biloba and increased the content of total flavonoids (Lu et al., 2022).

For a long time, most studies on the function of *SAUR* genes have focused on their key roles in regulating cell elongation, differentiation and auxin signal transduction in plants (Spartz et al., 2012; Kong et al., 2013; Huang et al., 2023). SAUR protein is also involved in the synthesis of secondary metabolites such as anthocyanins in plants (Kant et al., 2009; Hou et al., 2013). Sun et al. (2023) reported that GA_3_ can up-regulate the expression of *GmSAUR* gene, so we speculated that GA_3_-promoted *GmSAUR* gene is involved in flavonoid biosynthesis pathway. We found that the content of daizin and genistin in GA_3_-promoted *GmSAUR: GmSAUR* transgenic soybean was significantly higher than that in wild type (**Figure 2**). Further mining of ChIP-seq data of transgenic soybean GmSAUR showed that GmSAUR could bind to the *GmMYB176* promoter and regulate the mRNA level of *GmMYB176* (**Figure 3**). Studies have shown that *MYB176* is a transcription factor. Which belongs to the MYB family gene and participates in the metabolic pathway of plant flavonoids (Yi et al., 2010; Li et al., 2012; Gupta et al., 2019; Anguraj Vadivel et al., 2021). These results indicate that the *GmSAUR* gene may be involved in the flavonoid biosynthesis pathway.

Isoflavone is a complex quantitative trait, and its content in soybean may be affected by genotype, geographical location, climatic conditions and other factors. Soybeans contain four major isoflavones, namely aglycones, glycoside, acetylglycosides and malonylglycosides (Bennetts et al., 1946).Malonylglycosides and its derivatives are the main forms of isoflavones in soybean, accounting for 82.5% of total isoflavones (TIF). The contents of glycosides (Daidzin, Genistin, Glycitin), aglycones (Daidzein, Genistein, Glycitein) and acetyl glycosides (Acetyldaidzin, Acetylgenistin, Acetylglycitin) accounted for 16.48%, 0.88% and 0.81% of TIF, respectively (Sun et al., 2011; Liu et al., 2016; Wu et al., 2016; Azam et al., 2020). In soybean, daidzein, glycitein and genistein are three kinds of aglycone soybean isoflavones, which are directly synthesized by the plant phenylalanine pathway. Other types of soybean isoflavones are synthesized by glycosylation or malonylation after the metabolites are transported to the Golgi apparatus (Noguchi et al., 2007). Genistein and Daidzein are formed by Naringenin and Liquiritigenin catalyzed by IFS enzyme, respectively (Hashim et al., 1990). However, according to the latest research, the production of Glycitein is mainly based on daidzein. Daidzein forms 6-hydroxydaidzein through isoflavone 6-hydroxylase 1(IF6H1), and 6-hydroxydaidzein forms Glycitein under the action of isoflavone 6-O-methyltransferase(I6OMT) (Yang et al., 2025).Glycitein, daidzein and genistein are catalyzed by isoflavone 7-O-glucosyltransferase (IF7GT) to produce glycitin, daidzin and genistin, respectively (Noguchi et al., 2007). In this study, we found that the contents of daidzin and genistin in transgenic soybeans were significantly higher than those in wild-type (WT) plants, while the content of glycitin was significantly lower(**Figures 2C–E**). We speculate that the decrease of glycitin content may be related to the ‘ shunt effect ‘ of metabolic pathways. The synthesis of glycitein requires IF6H1 and I6OMT. If soybean varieties naturally contain low-activity IF6H1, the ability to convert daidzein to 6-hydroxydaidzein will be weakened. Knockout of *GmIF6H1* gene will significantly reduce the content of glycitin (Yang et al., 2025), so we speculate that *GmSAUR* may inhibit the expression of a specific IF6H1 gene and lead to a decrease in the content of glycitin in its grains. In the follow-up study, we will focus on the verification of enzyme gene expression in *GmSAUR* grains.

This study revealed that GmSAUR up-regulated its expression by directly binding to the promoter of *GmMYB176*. In the absence of GA_3_, *GmMYB176* activated the transcription of a series of isoflavone biosynthetic genes, such as *GmIF7MaT*, *GmC4H*, and *GmCYP450*. The up-regulated expression of these genes directly led to the significant accumulation of core isoflavone compounds-daidzin and genistin. However, in the presence of GA_3_, GA_3_ up-regulated the biosynthesis of isoflavones by inducing the expression of *SAUR*. Since *GmMYB176* is insensitive to GA_3_ signaling and may not be involved in the gibberellin signaling pathway (**Figure 3F**), we speculate that GA_3_ promotes the synthesis of isoflavones in *GmSAUR* transgenic soybeans may depend on other pathways (**Figure 7**). Although the involvement of gibberellin signaling in the regulation of flavonoid levels has been confirmed (Tan et al., 2019; Li et al., 2020a), more molecular regulatory mechanisms need to be further studied. This discovery not only expands the functional spectrum of *SAUR* genes, but also directly links the classic *SAUR* (*small auxin-up RNA*) genes to the synthesis of secondary metabolites isoflavones, providing a new perspective for understanding plant secondary metabolism. In the future, it will be important to elucidate the molecular mechanism of flavonol biosynthesis triggered by external environmental stimuli. However, we have not yet directly proven that *GmSAUR* is the “sole” or “key” effector downstream of GA signaling regulating isoflavones. Other GA-responsive factors may also be involved. Further research on transcription factors that regulate the interaction between hormones and flavonoids is expected to bring new breakthroughs in the mechanism of these hormone interactions.

## Data Availability

The Phytozome database contains the sequencing data covered in this research with accession numbers: *GmSAUR* (Glyma.06G281200), *GmMYB176* (Glyma.05G032200), *GmC4H* (Glyma.20G114200), *GmIF7MaT* (Glyma.19G030500), *GmCYP450* 84A1-like (Glyma.01G169200), *GmCYP450* 84A1 (Glyma.16G131200), *GmCHS8* (Glyma.11G011500), *GmIFS1* (Glyma.07G202300), GmIFS2 (Glyma.13G173500), *Gm4CL* (Glyma.13G323000), *GmPAL* (Glyma.10G058200). The *GmMYB176* RNA-seq data we used is stored in the NCBI-SRA database, and their storage location and accession number can be found under the BioProject number. PRJNA1255410 and accession nos. SRR33322692, SRR33322693 SRR33322694, SRR33322695, SRR33322696, and SRR33322697. The following is a list of accession numbers and names for the *GmSAUR* ChIP-seq data repository: NCBI-SRA database under BioProject PRJNA903963 with accession numbers SRR22387718, SRR22387719, and SRR22387720., and SRR22387721.The *GmSAUR* metabolome raw data can be found below: MetaboLights accession:MTBLS12627.

## Supplementary material

Supplementary Table S1 The specific sequences of the primers and free probes.

Supplementary Figure S1 Identification of *GmSAUR: GmSAUR*(mRNA), *35S:GmMYB176* and *myb176-c* lines.

Supplementary Figure S2 Subcellular localization of the GmMYB176 protein.

Supplementary Figure S3 RT-qPCR was employed to assess the expression of isoflavone synthesis genes in the *GmSAUR: GmSAUR* and the wild type.

Supplementary Data S1 Quantitative results of target metabolites between *GmSAUR* and WT

Supplementary Data S2 DEGs between *GmMYB176* and the empty vector by RNA-Seq.

Supplementary Data S3 GmSAUR ChIP-seq sequence alignment to the reference genome.

Supplementary Data S4 QC sample correlation of *GmSAUR* metabolomics.
