# Supplementary material for: Small auxin-up RNA promotes GmMYB176 gene transcription to modulate seed isoflavone accumulation in soybean
Source: Front Plant Sci. 2026 May 7;17:1841460. doi: 10.3389/fpls.2026.1841460 (PMC13190601; doi:10.3389/fpls.2026.1841460)
Supplement: Supplementary Table S1 — The specific sequences of the primers and free probes. [file DataSheet1.docx]

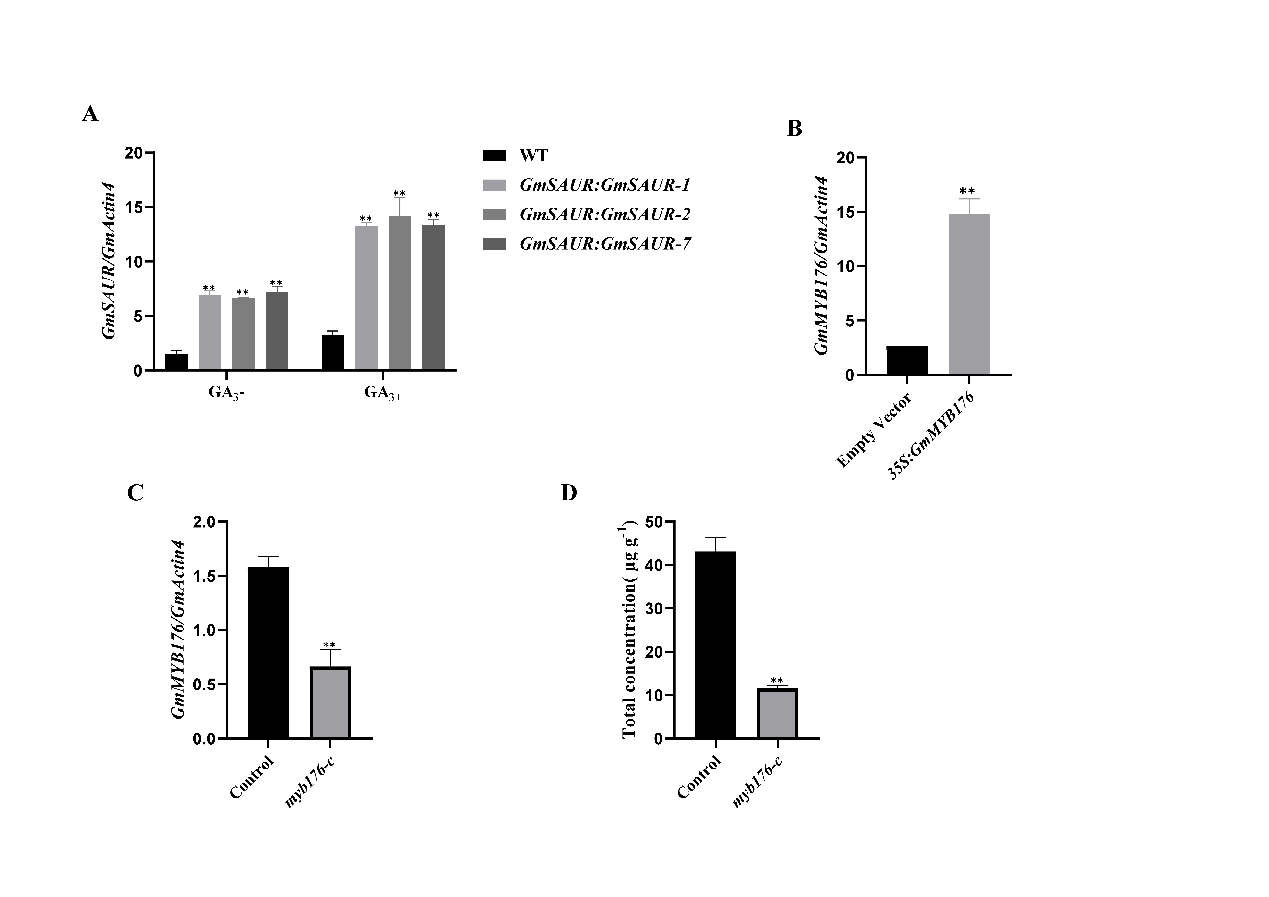


Supplemental Figure S1 Identification of *GmSAUR:GmSAUR* (mRNA), *35S:GmMYB176* and *myb176-c* lines.

(A):Relative expression of the *GmSAUR* of *GmSAUR: GmSAUR* and WT, respectively. Values are means±SD. Three biological replicates were performed. The asterisks indicate a significant change compared to the control. Two-tailed Student’s t-test, **P < 0.01.

(B):Relative expression of the *GmMYB176* of *35S:GmMYB176* and empty vector, respectively. Values are means±SD. Three biological replicates were performed. The asterisks indicate a significant change compared to the control. Two-tailed Student’s t-test, **P < 0.01.

(C):Relative expression of the *GmMYB176* of *myb176-c* and control, respectively. Values are means±SD. Three biological replicates were performed. The asterisks indicate a significant change compared to the control. Two-tailed Student’s t-test, **P < 0.01.

(D): Effects of *GmMYB176* silencing on isoflavonoid levels in soybean hairy roots. Three biological replicates were performed. The asterisks indicate a significant change compared to the control. Two-tailed Student’s t-test, **P < 0.01.


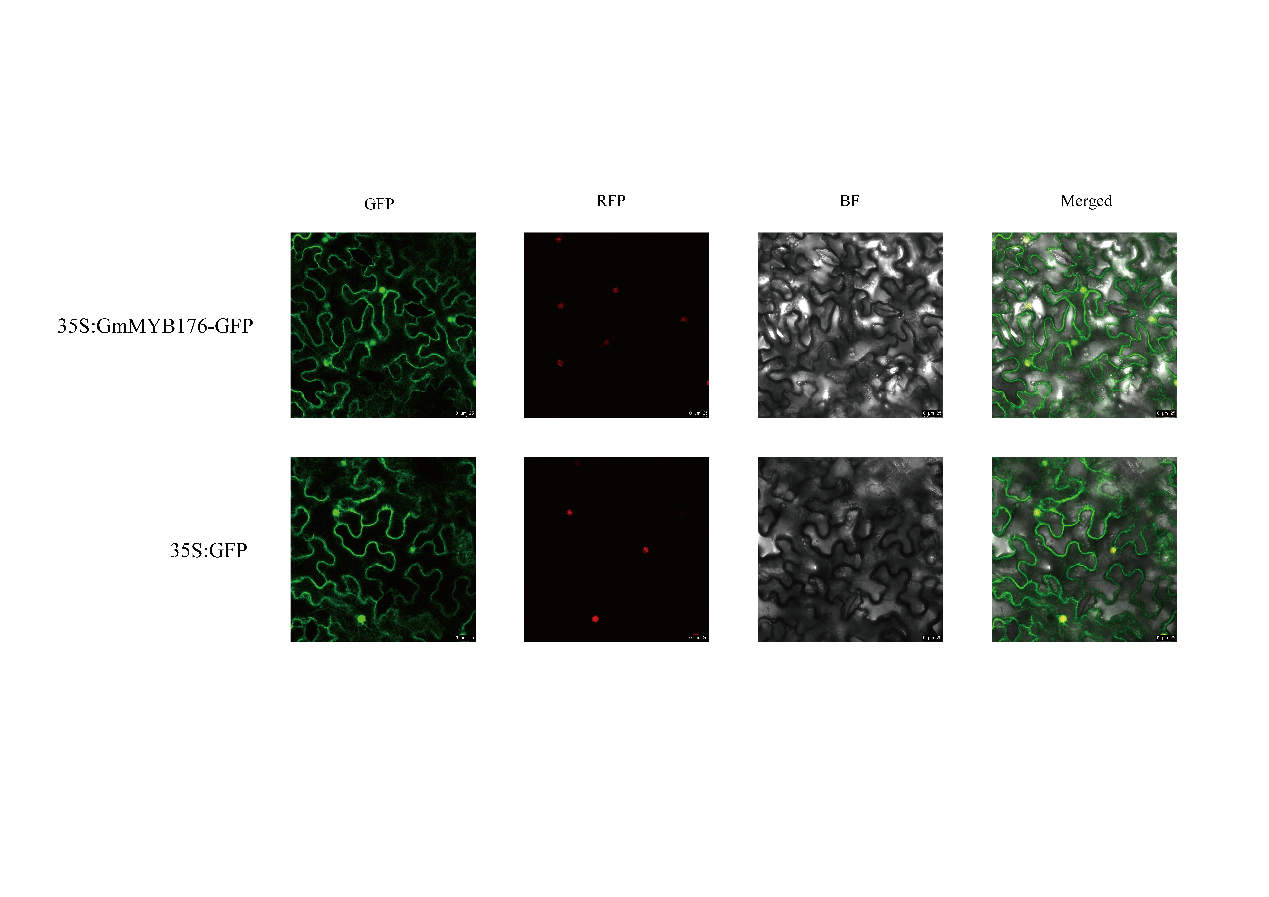


Supplemental Figure S2 Subcellular localization of the GmMYB176 protein.

A red nuclear marker plasmid (H2B-RFP) was used to confirm the location of the cell nucleus. GFP, green fluorescent protein; RFP, red fluorescent protein; BF, bright field; Merge, GFP, RFP, and bright-field images. Scale bars=25μm.


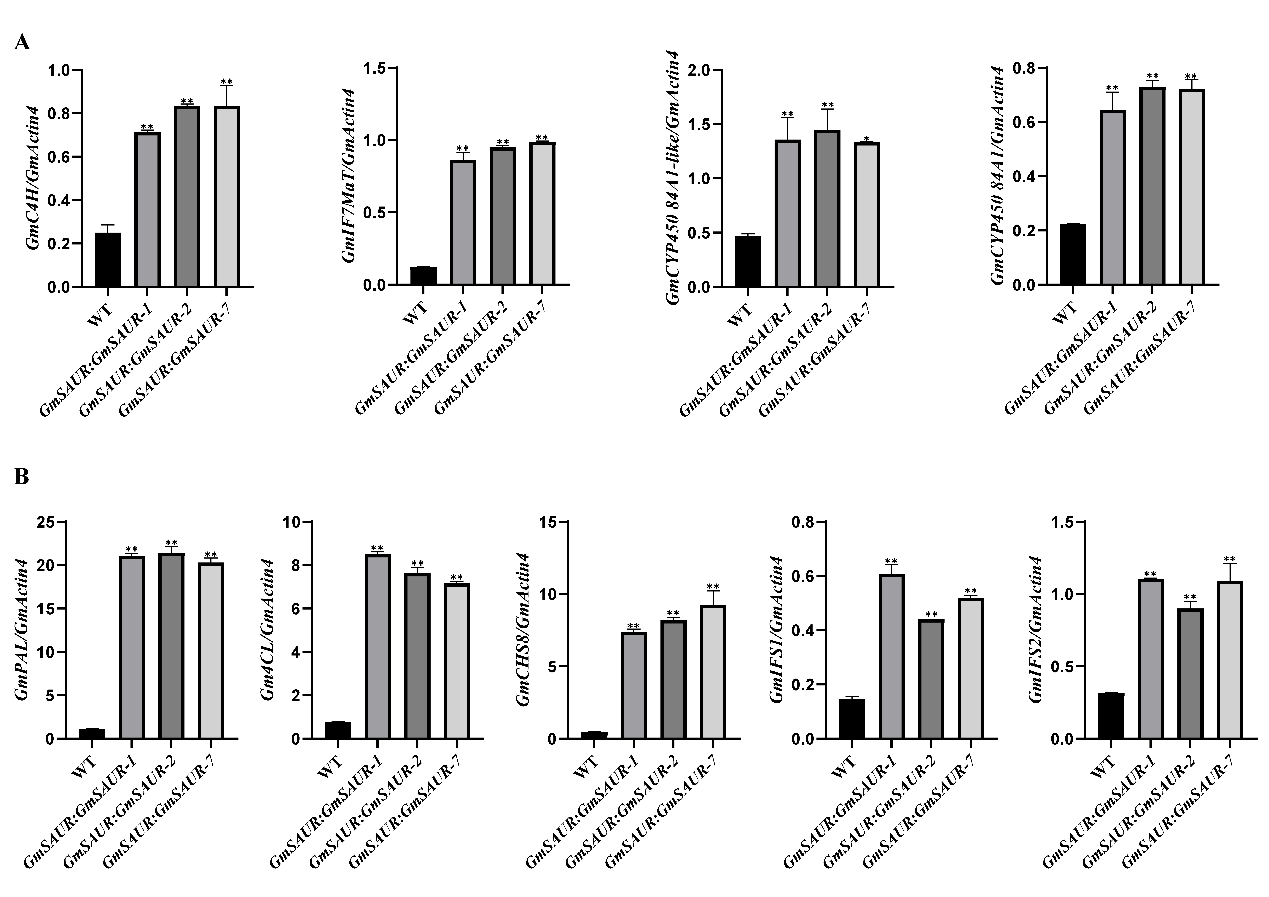


Supplemental Figure S3 RT-qPCR was employed to assess the expression of isoflavone synthesis genes in the *GmSAUR: GmSAUR* and the wild type.

(A) The relative expression levels of *GmC4H, GmIF7MaT, GmCYP450 84A1-li*ke, and *GmCYP450 84A1* *in GmSAUR: GmSAUR* and the wild type plants, respectively.

(B) The relative expression levels of *GmPAL*, *Gm4CL, GmCHS8, Gm*IFS1, and *GmIFS2 in GmSAUR: GmSAUR* and the wild type plants, respectively.

Each experiment included three technical replicates. The data were displayed as mean ± standard deviation (SD). These values were calculated based on the results of three independent replicates of experiments. Asterisks indicate significant differences between *GmSAUR:GmSAUR*-transgenic plants and wild type (**P* < 0.05, ***P* < 0.01; One-way ANOVA).

**Supplemental** **Table S1.** **The specific sequences of the primers and free probes.**

| Primer | Sequence (5’-3’) |
| --- | --- |
| *GmMYB176:LUC-*F | GAATTCCTGCAGCCCATCCCGGATATATTGAGGCA |
| *GmMYB176:LUC-*R | ACTAGTGGATCCCCCGAGTCCGTACGAGTCGAG |
| *qGmMYB176*-ChIP*-*F | TGGACAGGAAACAAGGATAAAG |
| *qGmMYB176*-ChIP-R | AGAAGGAAGTGAAAGAAAATGT |
| *GmMYB176-TOPO-*F | CACCATGTCTCGCGCCTCTTCCGCCG |
| *GmMYB176-TOPO-*R | TCAAGCAACACTAATGATGCTA |
| GmMYB176 Free probe-F | [biotin] GACTAAGTTGAAGATGCAGTTGCTATACAACCTCTCATTCAAGTAC |
| GmMYB176 Free probe-R | GTACTTGAATGAGAGGTTGTATAGCAACTGCATCTTCAACTTAGTC |
| *qGmActin4-*F | GTGTCAGCCATACTGTCCCCATTT |
| *qGmActin4-*R | GTTTCAAGCTCTTGCTCGTAATCA |
| *qGmMYB176-*F | AAGGAAAGGGCGATGGATTAGA |
| *qGmMYB176-*R | CCAACTGAAAGAGCCACAAAGA |
| *qGmSAUR*-F | TATACAACCCAAGCAGCCTCCA |
| *qGmSAUR*-R | AACCGTCTCATCTTATCTCCAACA |
| *qGmC4H-*F1 | GAACCCAGAGGAGTTCAGGC |
| *qGmC4H-*R1 | AATCTTTGTCCCCGCTGGAG |
| *qGmIF7MaT-*F1 | CCGGGAACGTACTTTGGGAA |
| *qGmIF7MaT-*R1 | CAGAGAACAACCTCGGCCTC |
| *qGmCYP450 84A1-like-*F1 | CAATAGCACTCCTGGTGGCG |
| *qGmCYP450 84A1-like-*R1 | TGGACTTGGAGAACCTGACG |
| *qGmCYP450 84A1-*F1 | TCGGGTCTAGTTCCCAGGAG |
| *qGmCYP450 84A1-*R1 | ATGAAGCTATCCAGCGAGGC |
| *qGmCHS8*-F1 | CTCCAGACAGTGAAGGTGCTAT |
| *qGmCHS8*-R1 | CAATGTTCTTTGAGACAATCCC |
| *qGmIFS1*-F1 | AAACTCGGGATCACAGAAACC |
| *qGmIFS1*-R1 | GGACGCAAGTGCAGAAACAA |
| *qGmIFS2*-F1 | ACCAAGGACGAGAACACGATG |
| *qGmIFS2*-R1 | GCTTGGTGGGTTTGGGAGA |
| *qGm4CL*-F1 | TACGGGAATGCTTGTCGCC |
| *qGm4CL*-R1 | CGTACCCATATCTCCCCCAACT |
| *qGmPAL*-F1 | TAGGGGAACAATCACAGCATCT |
| *qGmPAL*-R1 | TCACCAGAGGGTCCAACAGC |

**S****upplemental Table S2.** Q30 value of GmMYB176 RNA-seq.

|  | EV1 | EV2 | EV3 | MYB176-1 | MYB176-2 | MYB176-3 |
| --- | --- | --- | --- | --- | --- | --- |
| Total Reads Count(#) | 60218590 | 58366574 | 58496740 | 61834218 | 57869300 | 57962700 |
| Total Bases Count(bp) | 9032788500 | 8754986100 | 8774511000 | 9275132700 | 8680395000 | 8694405000 |
| Q30 Bases Count(bp) | 8583871549 | 8296374689 | 8333490408 | 8824726172 | 8241486543 | 8258393642 |
| Q30 Bases Ratio(%) | 95.03% | 94.76% | 94.97% | 95.14% | 94.94% | 94.99% |
